# Reusing wasteroot of *Rubia wallichiana* dyeing from Monpa of Tibet in China

**DOI:** 10.1038/s41598-021-93848-8

**Published:** 2021-07-12

**Authors:** Rong Yang, Yu Zhang, Sailesh Ranjitkar, Mingxiang Li, Yongjie Guo, Xiuxiang Yan, Chuanfa Wang, John Richard Stepp, Lixin Yang

**Affiliations:** 1 Southwest Forestry University, Kunming, 650224 Yunnan China; 2 Key Laboratory of Economic Plants and Biotechnology, Kunming Institute of Botany, Chinese Academy of Sciences, Kunming, 650201 Yunnan China; 3 N.Gene Solution of Natural Innovation, Kathmandu, 44614 Nepal; 4 Mid-Western University, Faculty of Humanities and Social Science, Lalitpur, 44700 Nepal; 5 Lumbini Buddhist University, Faculty of Agroforestry, Lumbani, Nepal; 6 Center for Biodiversity and Indigenous Knowledge, Kunming, 650034 Yunnan China; 7 State Key Laboratory of Phytochemistry and Plant Resources in West China, Kunming Institute of Botany, Chinese Academy of Sciences, Kunming, 650201 Yunnan China; 8 Ethnobiology Lab, Department of Anthropology, University of Florida, Gainesville, FL 32611-7305 USA; 9 Germplasm Bank of Wild Species of China, Kunming Institute of Botany, Chinese Academy of Sciences, Kunming, 650201 Yunnan China; 10 University of Chinese Academy of Sciences, Beijing, 100049 China

**Keywords:** Biological techniques, Biotechnology, Plant sciences, Environmental sciences

## Abstract

Humans have dyed textiles and leather, colored food, and paint body parts using natural dyes throughout history. Natural dyes have suffered drastically due to recent socioeconomic changes and replacement with synthetic dye in the textile industry. Hence, it is urgent to study indigenous dye plants and dyeing craft in local communities to protect these resources' potential ecological, economic, and cultural values. In 11 Monpa villages of Mêdog County, South-east Tibet, China, we conducted field research to record the indigenous method of cloth dyeing using madder dye. An aqueous extract of the root of *Rubia wallichiana* is a traditional madder dye. In this study, we used traditional dye and ethyl alcohol extract of the residue of aqueous extract. Two fabrics were dyed with the extractions in the presence of one of the metallic mordants or biomordants. Pigment compounds from aqueous extraction of madder and ethyl alcohol extraction of recycled madder were evaluated using Phytochemical, UV–visible spectroscopy, and FTIR test. We carried out One-way ANOVA and Duncan's new multiple range method to analyze different dying approaches and conditions. The dyed fabrics were evaluated by indicators of color strength and fastness, including washing, rubbing, and perspiration. The findings revealed the potentiality of biomordants to improve the dyeing properties of madder. The dyeing properties of recycled madder were marginally better than traditional madder. The results revealed the feasibility of enhancing the dyeing property and reuse of the residue from madder dyeing. The improved dyeing and reuse of residue can improve local ecological, economic benefits, and cultural heritage while applying research findings for the subsequent commercialization of plant dyes.

## Introduction

An adequate understanding of the local techniques is essential to get the proper benefit and sustainable use of rich natural resources. Dye-yielding plants can contribute to economic development, industrialization, and contributing to people's livelihood improvement. However, the improvement of the plant dyeing industry would be beneficial to environmental and cultural conservation.

The textile industry is now one of the essential sectors globally, contributing to a significant share of annual global economic output. In recent decades, textile and clothing production has seen high growth rates and increased international trade^1^. Many Asian countries are the world’s leading textile exporters and this support these countries' rapid economic growth^2^. Along with this development, China is now the largest manufacturer and exporter of textiles worldwide^3^. China’s textile industry is one of the traditional pillars of the Chinese economy^4^.

Although economically significant, the fabrication of textiles is one of the largest sources of greenhouse gas emissions, requiring high-energy consumption leading to high levels of pollution^5^. The textile industry uses large amounts of freshwater along its entire value chain, particularly during dyeing, for dissolving dyes and chemicals^6^. Furthermore, synthetic dyes have a distinct environmental impact, and discharging wastewater creates major pollution issues^7^. Synthetic dyes not only pollute the environment and damage ecosystems but can also be harmful to human health^8,9^.

Thus, an “eco-efficiency” concept has come into existence to bring economic and environmental viability together^10^. As a biodegradable and recyclable resource, natural dyes are gradually beginning to receive more attention^11^, and widely planted industrial crops are a prime source for natural pigments^12^. In recent years, the frequency of using natural dyes has led to more sustainable and eco-friendly textile dyeing^13,14^. Natural dyes are extracted from natural resources, especially plants^15,16^, and the process relies on the vast cultural heritage of natural dyeing throughout the world^17,18^. Plant dyes are now becoming popular in the dyeing of cotton^19^, hemp^20^, silk^21^, and wool fabrics^22^. Also, plant dyes are widely used in cosmetics, foods, medicines, clothing^23–25^. Dyeing with plant extracts is challenging. They possess some disadvantages such as less or even no adhesion towards the textile substrate, have lower yields and colorfastness, and higher cost, which has restricted their potential in industrial-scale applications^26^. However, mordants can help create a bond between dye and fabric fiber, making the color brighter and improve colorfastness^27^. Therefore, the compatibility of mordant with the coloring agent is important. A range of metallic mordants are available, but metallic mordants can produce heavy metal pollution^28^. In contrast, biomordants, obtained from minerals, chitosans, sol-gels, and plants^29^, can provide eco-friendly industrial production. The utilization of biomordants is gradually becoming a trend in sustainable ecological dyeing^30,31^. Therefore, it is helpful in further study biomordants used traditionally, such as the fruit of *Chaenomeles speciosa* (Sweet) Nakai^32^, and gum rosin^33^ that could improve the effect of plant dyeing.

Anthraquinone dyes from plant dye are relatively stable and lightfast. Madder (*Rubia spp.)* is an anthraquinone dye source and has been a popular source of red color shades and orange, gray, purple, pink, and brown shades^34^. *Rubia tinctorum* L. and *Rubia cordifolia* L. have been extensively used for dyeing throughout history. Alizarin and purpurin of anthraquinones compounds are among the valuable extract from *Rubia tinctorum* L. and *Rubia cordifolia* L.*,* respectively^13^. Few earlier works reported the dyeing properties of *Rubia tinctorum* L.^35,36^ and *Rubia cordifolia* L.^37–40^. However, no research on dyeing applications has explored the madder plant known as *Rubia wallichiana* D. In fact, it is an important dye-yielding plant, and still used by the Monpa in Mêdog County of the Tibet Autonomous Region in China. The colorant of madder is a cultural symbol for the Monpa which represents worship and kindness, vitality and beauty, truth and enthusiasm in their life. Due to the particularity of the geography, the distinctive biophysical environment allows for a rich plant diversity in the region. It also supports the traditional dyeing culture and traditional ecological knowledge of the Monpa people, who also live in the Indian state of Arunachal Pradesh while the majority of the Monpa population in Tibet is mainly concentrated in Mêdog County^41^. The Monpa people use the wild roots of *R. wallichiana* for dyeing through traditional practices. Traditional madder dyeing has some disadvantages, such as a sometimes incomplete fixing of dyes and pigments as well as limited availability in the wild. The root of the perennial plant is the only part used in extracting dye so its collection and production cost are high. Previous research on madder dyeing regarding environmental sustainability includes the secondary utilization of dye solution^11^ and energy reduction in dye extraction^42^. However, recycling the residue of used madder has not been explored.

Therefore, it is necessary to study and understand how to use the residue of used madder for the sustainable and complete use of these valuable resources. This study aims to document and assist in preserving traditional Monpa dyeing craft through improved techniques. The specific objectives were:

1. To expand knowledge on traditional Monpa madder textile dyeing;
2. To analyze the efficiency of recycled madder with biomordant dyeing agents;
3. To explore the chemical bonding of the plant dye, biomordants, and textiles.

## Methods

### Study site

**Figure 1:**
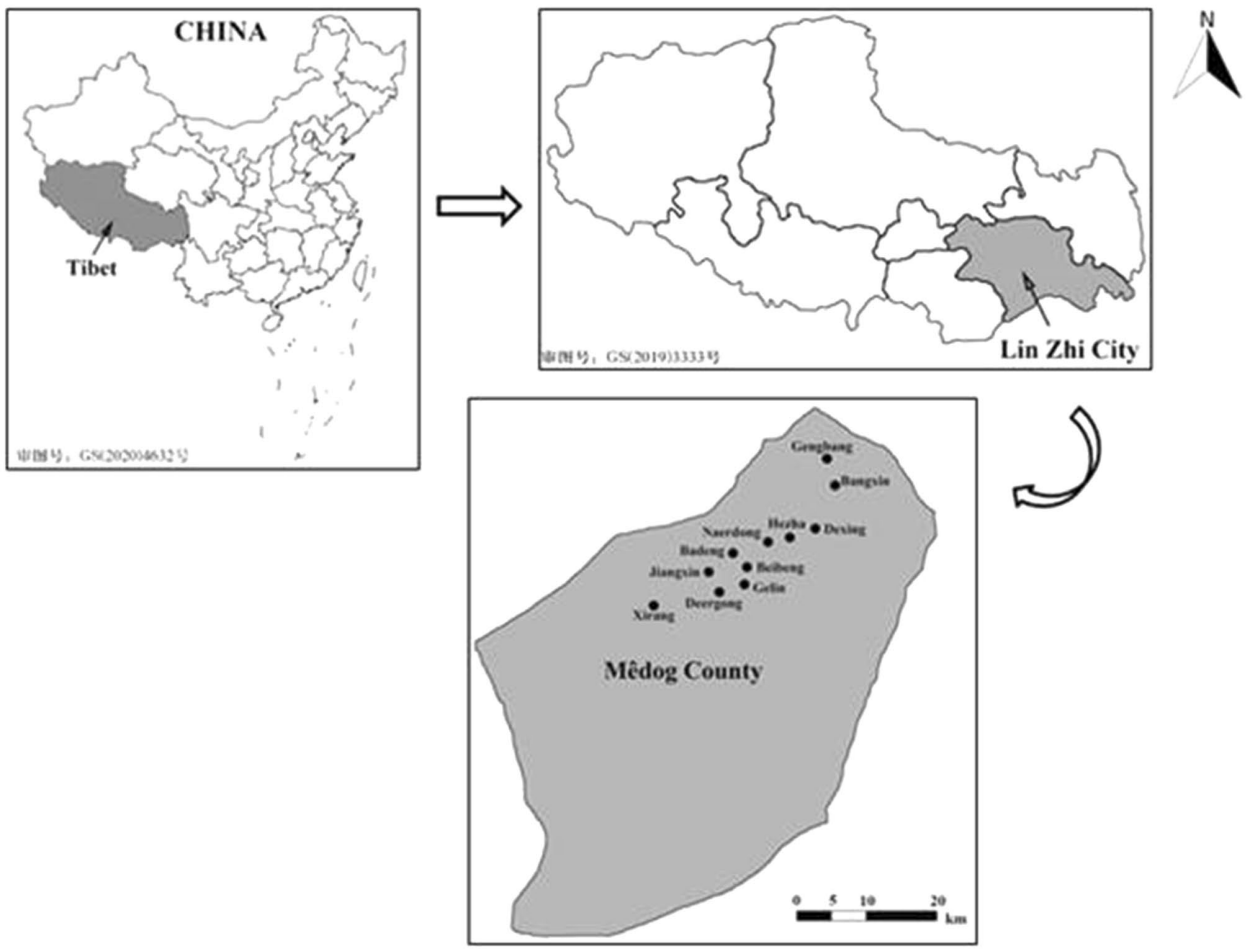
The location of 11 study villages in Mêdog County, Lin Zhi City, Tibet, China.

The Monpa of Mêdog County in south-eastern Tibet live at the edge of Himalayan and the Indo-Burma biodiversity hotspots. The area is famous as the “third pole of the earth”^43,44^. Once flourishing in this fragile ecosystem, Monpa traditional ecological knowledge has been diminished, although culturally important rare and endangered species persist in the region. Mêdog County is located in the lower reaches of the Yarlung Tsangpo River, which is in the southeast of Tibet with an area 31,395 km^2^. The annual average temperature in Mêdog County is between 16and 18 °C, the lowest temperature is 2 °C in January, and the highest temperature is 33.8 °C in July. The annual precipitation is 2350 mm, the frost-free period is about 330 days, and the average humidity is more than 80%^45,46^. Beibeng Township is the main settlement area of the Monpa people in the region and accounts for 99.2% of the total population of Monpa in China^47^. Mêdog County is the last county in the region to gain highway infrastructure and has had little outside interaction until recently. 11 communities were selected as the study sites in Mêdog County (Fig. 1). The map in Fig. 1 was made by Adobe Photoshop CS5 (http://bzdt.ch.mnr.gov.cn/). The research was carried out in four Townships, including Beibeng Township, Dexing Township, Bangxin Township, and Jiaresa Township.

### Field research

We used informant referral sampling to recruit a group of 45 informants, ages 18 to 85 years old, 25 women, and 20 men. Semi-structured interviews were conducted in August 2018 to document Monpa traditional knowledge of dye-yielding plants. The interview questions focused on seven questions: (1) When are dye-yielding plants collected? (2) What are the local names of the dye-yielding plants? (3) Where do dye-yielding plants grow in their community? (4) Which part of the dye-yielding plant do they mainly use for dyeing? (5) What are the processes for indigenous dye plant dyeing? (6) Do they add anything else to the plant dyeing process? (7) Do they mainly use dye-yielding plants for dyeing food or fabric?

This information was put into an ethnobotanical catalog which included a herbarium voucher code, Chinese name, scientific name, Monpa name, family name, life form, parts used, color, habitat, and medicinal value (Table 1). All voucher specimens were identified and verified by local Monpa people, botanists, and experts from the Kunming Institute of Botany.

**Table 1:** Dye plants used by Monpa communities in Mêdog County.

Herbarium voucher code	Chinese name	Monpa name	Scientific name	Family name	Parts used	Life from	Habitat	Color	Dye uses	Medicinal value
DX08	Jianghuang 姜黄	Jiong	*Curcuma longa* L	Zingiberaceae	Leaves, roots	Herb	Herb in plains, mountain meadows and bushes	Yellow, golden yellow, orange	Cloth, food	Treating chest pain, irregular menstruation, traumatic injury
BB08	Guangjingqiancao 光茎茜草	Lae-nyi	*Rubia wallichiana* Decne	Rubiaceae	Stems, leaves, roots	Herb	Herb under forest or thicket	Red	Cloth	Treating itchy skin
BG03	Hetao 核桃	Da ga xin	*Juglans regia* L	Juglandaceae	Peels	Tree	Tree in hillsides, valleys, rivers and forests	Brown	Cloth	Reinforcing kidney, Relaxing bowels
BB06	Banlan 板蓝	Yang xian ba	*Baphicacanthus cusia* (Nees) Bremek	Acanthaceae	Stems, leaves	Herb	Herb under the trees, by the stream	Blue, black	Cloth	Clear heat and detoxify, cool blood and reduce swelling
BB18	Yujin 郁金	Dgrong	*Curcuma aromatica* Salisb	Zingiberaceae	Roots, stem	Herb	Herb under the forest	Yellow	Cloth	Inducing menstruation to relieve menalgia
JR06	Jiang 姜	Sa-ga	*Zingiber officinale* Rosc	Zingiberaceae	Roots, stems	Herb	Herb in thickets, hillside forests	Yellow	Cloth, food	Treating cold
BB16	Ruichifengxianhua 锐齿凤仙花	Zhang zeng mu n	*Impatiens arguta* Hook	Balsaminaceae	Flowers	Herb	Herb under the forest, in wet places, and near the ditch	Red	Cloth, nails	Treating traumatic injury
DX02	Sang 桑	Sen lin xin	*Morus alba* Linnaeus	Moraceae	Fruits	Shrub	Tree in the forest	Red	Cloth	Eliminating stasis to subdue swelling, clearing away the lung-heat, improving eyesight
BG07	Banli 板栗	Cai xin se	*Castanea mollissima* Bl	Fagaceae	Peels, leaves	Herb	Tree in the low hills gentle slope and river beach area	Yellow, brown, black	Cloth, food	Nourishing the stomach and spleen, tonifying kidney
JR13	Shanglu 商陆	Mini gan bu ong	*Phytolacca acinosa* Roxb	Phytolaccaceae	Fruits	Herb	Herb by the side of the road and behind the house	Purple,red	Cloth	Treating carbuncle swollen sore poison
BG12	Hezi 诃子	Ga xia tong xin	*Terminalia chebula* Retz	Combretaceae	Peels, barks	Tree	Tree in the forest	Yellow, brown	Cloth	Treating of chronic Dysentery
DX09	Weijianyeling 尾尖叶柃	Zem-shing	*Eurya acuminata* DC	Pentaphylacaceae	Leaves	Shrub	Shrub on a hillside in a forest or thicket	Yellow, green	Cloth	Clear heat and detoxify
JR15	Jinxiancao 金线草	Lae-nyi	*Rubia membranacea* Diels	Rubiaceae	Stems	Herb	Herb in forests, forest margins, thickets, or grasslands	Orange, red	Cloth	Treating of injury pain, fracture, stomach pain, traumatic bleeding
BG10	Zisu 紫苏	Lang	*Perilla frutescens* (L.) Britt	Labiatae	Leaves	Herb	Herb under the hardwood forest on the hillside, or homegarden	Yellow green, green, Purple red	Cloth, food	Treating cold
DX05	Duying 杜英	A ru	*Elaeocarpus decipiens* Hemsl	Elaeocarpaceae	Barks	Tree	Tree in mountain and rainforest	Brown	Cloth	Treating falls and injuries

### Data analysis

We evaluated the cultural significance of each dye plant following Tardío and Pardo-de-Santayana^48^, using the following equation:

$${\text{CI}}_{{\text{S}}} = \sum\limits_{{{\text{u}} = {\text{u}}_{1} }}^{{{}^{{\text{u}}}{\text{NC}}}} {\sum\limits_{{{\text{i}} = {\text{i}}_{1} }}^{{{}^{{\text{i}}}{\text{N}}}} {\frac{{{\text{UR}}_{{{\text{ui}}}} }}{{\text{N}}}} }$$

N represents the total number of informants; NC represents the sum number of applications, for the species of dye plant S; UR_ui_ is a utilization report (UR) for the species S referred by informant in application u; Therefore, the CI represents the total proportion of informants that referred each type of the use of a given species.

This index reveals the diversity of use of each plant species and the degree of recognition of certain plant species by informants. The high CI value illustrates that the plant has many applications and most people identified it.

Data analysis of this study were used by One-way ANOVA and Duncan's new multiple range method^49^. Standard deviation were determined using significant difference test (*P* < 0.05). Statistical analyses were performed by SPSS 22 (Statistical Product and Service Solutions) and Excel 2007.

### Materials

We conducted an ethnobotanical survey to document dye-yielding plants and related Monpa traditional knowledge. The roots of *R. wallichiana* (madder) were collected in Autumn 2018 from De'ergong Village (29° 13′ 43″ N, 95° 08′ 11″ E), Beibeng Township of Mêdog County, Lin Zhi City, South-east Tibet, China. ‘Recycled madder’ is the residue madder root that remains after the collection of aqueous extract. Two biomordants, including the fruit of *C. speciosa* and gum rosin, were collected from the local period market in Heqing County, Dali Bai Autonomous Prefecture of China. Metallic mordants including alum, stannous chloride, and ethyl alcohol used were all in analytical reagent grade. Esquel Enterprises Ltd provided bleached cotton and hemp fabrics.

### Laboratory experiments

#### Extraction of plant dye

The roots of the madder were water-washed to remove impurities and cut into segments. Then, 100 g of the madder roots were put into 2.5 L of distilled water and boiled for 30 minutes^13,50^. After cooling, the solution was filtered, and the filtrated extract was diluted with water to 2.5 L. The resultant solution was the dye solution of the madder. Subsequently, the residue roots of madder were extracted again by the mixture of 1.5 L ethyl alcohol and 1.0 L distilled water at 60 °C for 30 minutes^51^. The resultant solution was the dye solution of recycled madder.

#### Dyeing

4% madder was used to dye cotton (weight 118.7 g m^−2^, plain weave) and hemp (weight 149.1 g m^−2^, plain weave) fabrics at 80 °C for 30 min with a bath ratio of 30:1 with a laboratory method. After dyeing, the dyed samples were rinsed with tap water and dried at room temperature. This dyeing process was based on Monpa traditional madder dyeing practices derived through field interviews.

#### Mordanting methods

Pre, simultaneous, and post-mordanting methods were implemented at 80 °C for 30 min at a liquor ratio of 30:1 by two metallic mordants (10% owf alum and stannous chloride) and two biomordants (20 g L^−1^ *C. speciosa*, 12 g L^−1^ gum rosin).

#### Color measurements

We estimated the color characteristics that include color difference (∆E*), lightness (L*), redness-greenness (a*), and blueness-yellowness (b*) of dyed samples. Benchtop spectrophotometer (Shenzhen ThreeNH Technology Co., Ltd; illuminant D65 and 10° standard observer) was used for color characterization. The standard samples were undyed fabrics. *K/S* was calculated by Kubelka–Munk equation:

$$K/S = (1 - R)^{2} /2R$$

where *R* is the reflectance of the dyed fabric, *K* is the absorption coefficient, and *S* is the scattering coefficient.

#### Colorfastness tests

We examined the colorfastness of dyed samples using Chinese Textiles Test Specification to washing, perspiration, and rubbing before and after mordanting as a standard. The colorfastness to washing, perspiration and rubbing was measured according to the standard of GB/T3921-2008, GB/T3922-2013, and GB/T3920-2008^52–54^ relatively, which are based on ISO international standards. The standardization uses grading on a scale of 1–5, where value 1 is poor and value 5 is excellent. Value 3 is assumed as a basic standard, with a grade of 4 and above considered acceptable for commercial use.

### Phytochemical analysis

Borntrager reaction is a special chromogenic experiment designed to detect hydroxyl anthraquinone components in plants. It is based on the characteristics of hydroxyl anthraquinone and its glycosides showing red or purplish-red in alkaline solution^55^. 100 mg of aqueous extract was used to analyze, 2 ml of diethyl ether, 1 ml of 5% sodium hydroxide, and 5 ml of 10% concentrated sulphuric acid was used to analyze the compound present in the aqueous extract in this study.

### FTIR and UV–visible analysis

We examined the absorption spectrum of madder extracts in the 190 to 600 nm wavenumber range using a UV-5500PC spectrophotometer (Shanghai Metash Instruments Co., Ltd). Fourier transform infrared spectrometer (Nicolet iS10, ThermoFisher Scientific) confirmed the FT-IR spectra of madder extract, with 16 scans at 4 cm^−1^ resolution in the spectral range of 4000–400 cm^-1^. The method of KBr pellet was used for spectrum data^16^.

### Ethics approval and consent for publication

All experimental protocols were approved by the Kunming Institute of Botany in Yunnan, China. We requested permission from the local community committee for fieldwork. We also explained our research and objectives to community leaders and requested assistance from them. All of our field studies were carried out with prior informed consent and followed relevant guidelines and regulations. Moreover, this study did not cause any harm or risk to participants. We also obtained informed consent for publication of identifying information/ images in online open-access.

## Results

### Ethnobotanical study of the Monpa dye plants

#### The diversity of dye plants in Monpa communities

As shown in Table 1, 15 plant species belonging to 12 plant taxonomic families were recorded as dye plants in the study area. The species were mainly herbaceous (10), while the remaining were trees (3), shrubs (2). The plant parts used for extracting pigment included leaves (6), stems (5), roots (4), peels (3), fruits (2), barks (2), and flowers (1). Various colors were produced from the 15 dye plants, such as red, yellow, blue, black, green, purple and so on. Dye plants were used to dye cloth, food, and also occasionally paint nails. These dye plants occurred in diverse habitats, and most of them also possess medicinal properties.

#### Evaluation of indigenous knowledge on dye plants

The CI value was used as a quantitative indicator to evaluate 15 species of dye-yielding plants. It was sorted by the size of the indicators (Table 2), *Rubia wallichiana* Decne. (2.11), *Baphicacanthus cusia* (Nees) Bremek. (2.04), *Juglans regia* L. (2.00), *Curcuma longa* L. (1.96), *Zingiber officinale* Rosc. (1.89), *Rubia membranacea* Diels. (1.80) revealed higher CI values, and the CI value of *R.wallichiana* was the highest. A high CI value indicates that the species has many uses, and the respondents have a deep understanding of plant utilization. It also shows that plants have high cultural importance to the Monpa. The use of *R.wallichiana* is of great significance in the region. *Phytolacca acinosa* Roxb. (0.84), *Elaeocarpus decipiens* Hemsl. (0.69), *Eurya acuminata* DC. (0.44) have lower CI values. The lower CI value possibly illustrates that these dye plants have few other uses.

**Table 2:** Cultural importance index of dye plants used by Monpa people.

Scientific name	The type of use			CI
	Dye	Food	Medicine	
*Rubia wallichiana* Decne	42	13	40	2.11
*Baphicacanthus cusia* (Nees) Bremek	39	14	39	2.04
*Juglans regia* L	35	40	15	2.00
*Curcuma longa* L	35	28	25	1.96
*Zingiber officinale* Rosc	30	45	10	1.89
*Rubia membranacea* Diels	18	38	25	1.80
*Castanea mollissima* Bl	23	38	18	1.76
*Perilla frutescens* (L.) Britt	20	22	25	1.49
*Impatiens arguta* Hook	38		28	1.47
*Curcuma aromatica* Salisb	18	11	35	1.42
*Morus alba* Linnaeus	18	20	25	1.40
*Terminalia chebula* Retz	13	10	35	1.29
*Phytolacca acinosa* Roxb	30		8	0.84
*Elaeocarpus decipiens* Hemsl	13		18	0.69
*Eurya acuminata* DC	20			0.44

#### The traditional knowledge of madder dyeing

The analysis and evaluation showed that the index of cultural importance on *R. wallichiana* was highest, so we conducted further research on this species. We found that indigenous madder dyeing was still in existence. The Monpa people maintained madder dyed clothes as their cultural identity. The madder is known locally as ‘Lae-nyi’ in the Monpa language, and it is mainly growing on the edge of the forest’s margins or shrubs at an elevation of 1500 m–3080 m. Monpa men collect the root of *R. wallichiana* each year during the Autumn season. We found two methods of dyeing cloth in Monpa communities. The first method was the direct dyeing method, mainly used to dye locally. The second method was an advanced one that use metallic salts to fix the color or obtain more colorants by the post-mordanting method. *R. wallichiana* is treated to retain only the roots, which are dried and cut into fragments. Subsequently, the madder pigment is extracted in boiling water for 30 min. Cotton or hemp yarns are shocked in boiling water for 10 min in another vessel to remove impurities. The dye liquid is then filtered and heated. The dyeing process includes shocking cotton or hemp yarn in hot extract for 30 min, repetitively stirring with a stick. The reason for this is to prevent the pigment from unevenly dyeing the yarns. The number of dyeing rounds depended on the depth of the desired color, and if the yarns are repeatedly dyed, they would show darker colors. Dyeing was followed by drying, and after that decision is made whether to add metallic salts. The dyed yarns are washed to remove the floating color, and then air-dried. Last, the dyed yarns are weaved into fabrics that are made into clothing.

### Color measurement evaluation

#### Comparison of madder with biomordants and metallic mordants

The findings of our experiment revealed that the color strength of madder dyed fabrics was higher when biomordants were used. The maximum K/S value was obtained in the dyed cotton and hemp (Table 3) fabrics with gum rosin in the post-mordanting. The minimum K/S value was obtained with stannous chloride in simultaneous mordanting. The second-highest K/S value was separately revealed in the dyed cotton and hemp fabrics with *C. speciosa* in the post and pre-mordanting methods. The results indicated that the maximum and second-highest K/S values were obtained with biomordants. Meanwhile, madder dyed fabrics with biomordants show higher K/S values than indigenous madder dyeing through the post mordanting method. The diverse colorimetric data confirmed the variation in the hue of the fabrics brought by different mordants and by different mordanting methods.

**Table 3:**
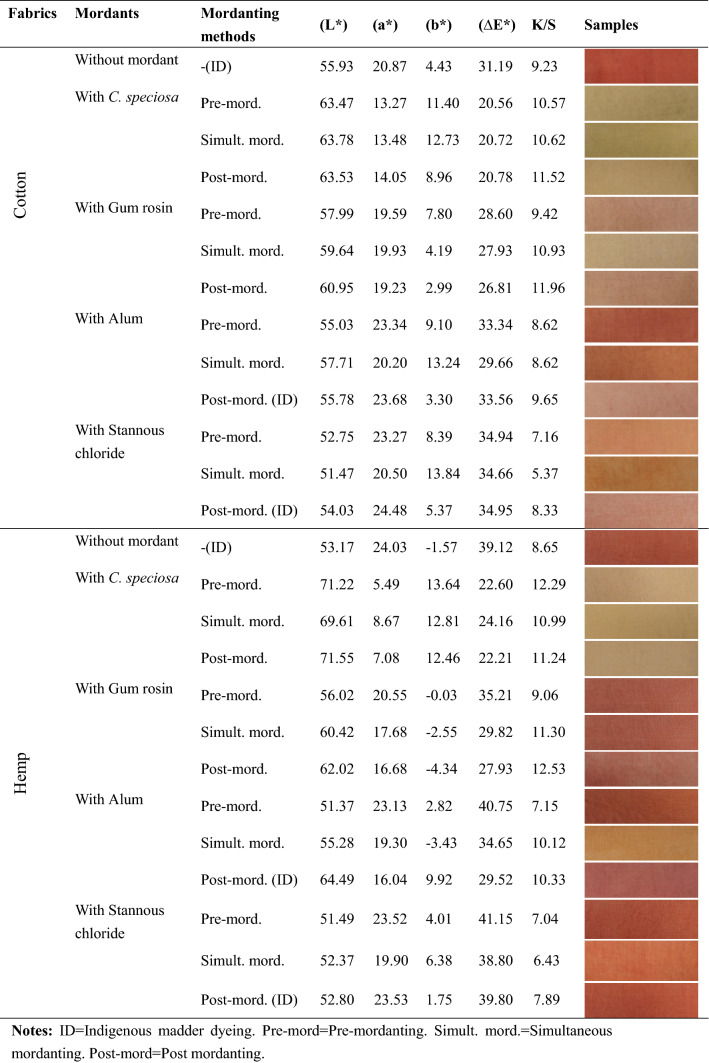
K/S values and colorimetric data of madder dyed fabrics with and without mordanting.

ID, Indigenous madder dyeing; Pre-mord, Pre-mordanting; Simult. mord., Simultaneous mordanting; Post-mord, Post mordanting.

It was observed that the K/S value of madder dyed fabrics with biomordants was higher than that of the metallic mordants in the same mordanting method. Our results indicated that biomordant could yield better results in cotton and hemp dyeing.The findings of this work are supported by research that recommends biomordants as an alternative to metallic mordants due to the superior properties^56^. .

#### Comparison of recycled madder with biomordants and metallic mordants

The K/S values of dyed fabrics under indigenous recycled madder dyeing methods were lower than those with biomordants in the same mordanting method. Dyed cotton and hemp (Table 4) fabrics showed the maximum K/S value with gum rosin in simultaneous mordanting, while the minimum K/S value was obtained without mordant. It was observed that the two fabrics yielded the maximum K/S values with biomordants. The findings indicated that biomordants were effective with recycled madder and performed superior to metallic mordant in three mordanting methods.

**Table 4:**
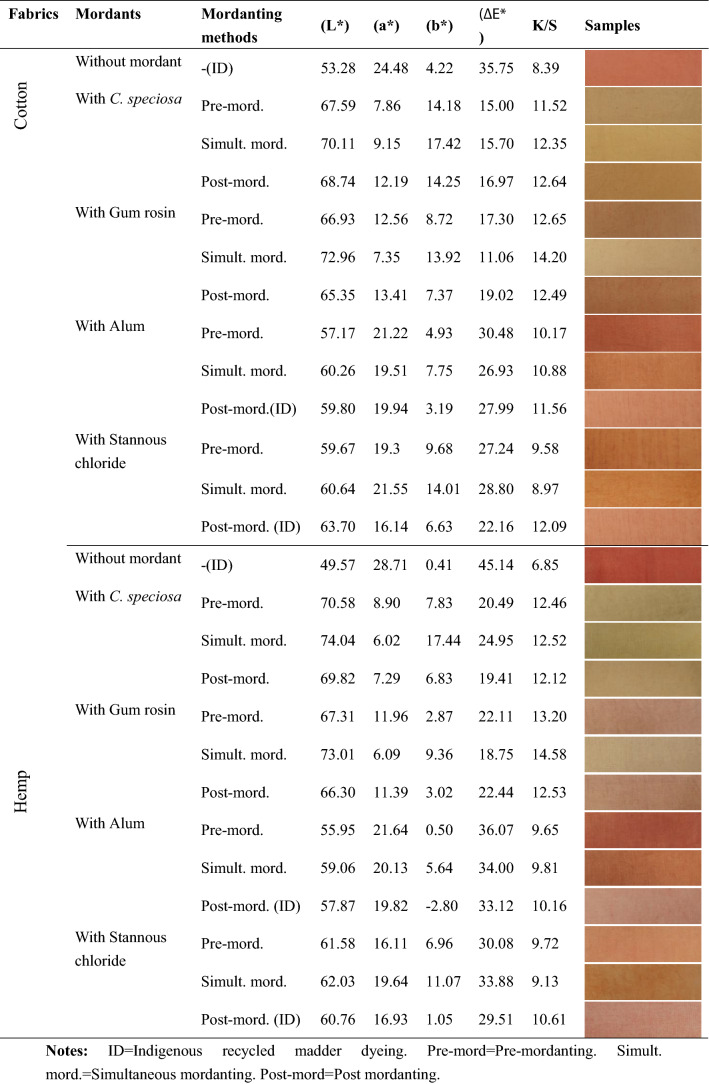
K/S values and colorimetric data of recycled madder dyed fabrics with and without mordanting.

ID, Indigenous recycled madder dyeing; Pre-mord, Pre-mordanting; Simult. mord., Simultaneous mordanting; Post-mord, Post mordanting.

#### Comparative effects on color strength of dyed fabrics between madder and recycled madder

**Figure 2:**
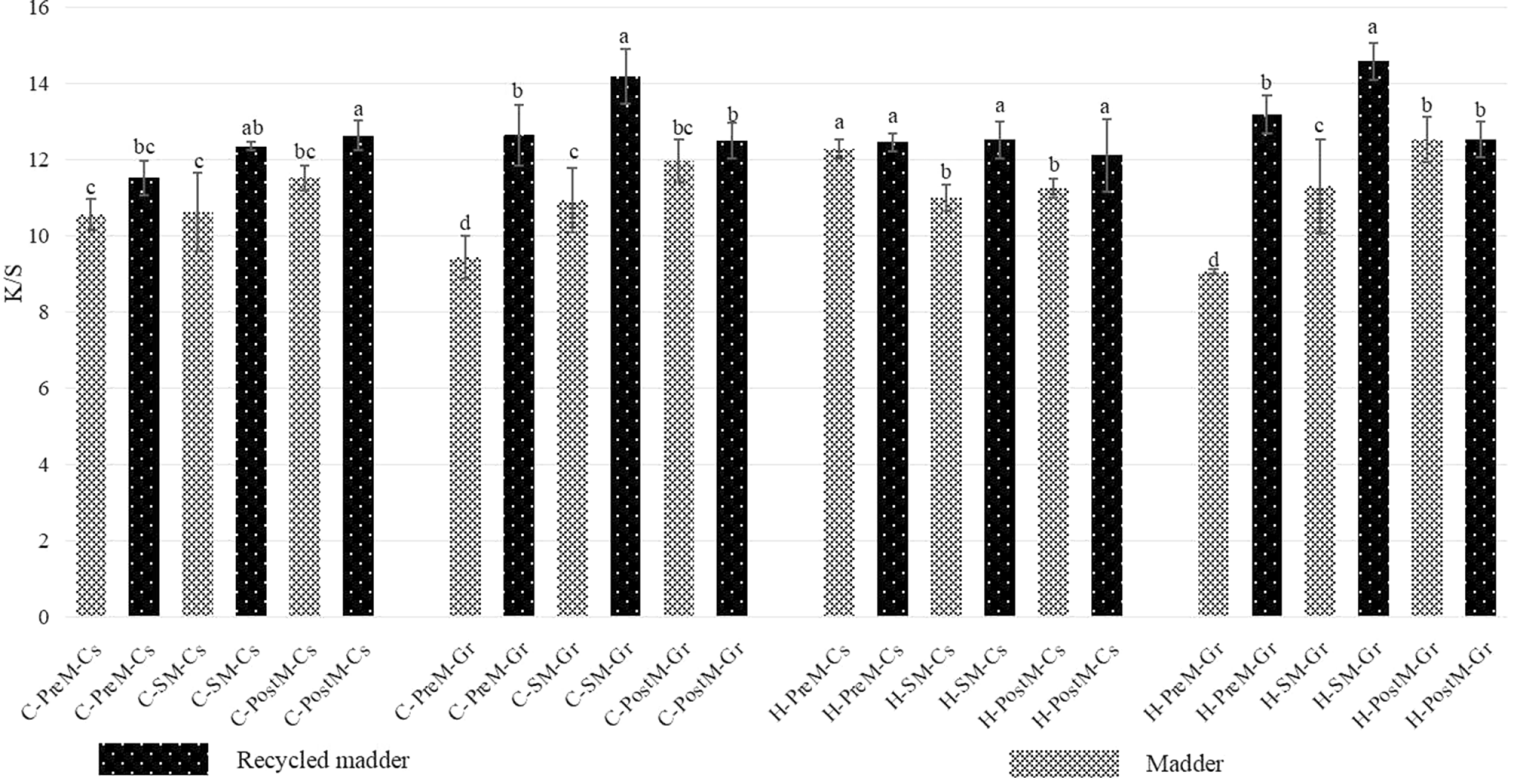
Comparison of color strength between madder and recycled madder with biomordants. (Biomordants: Cs = *C. speciosa*, Gr = Gum rosin; Fabrics: C = Cotton, H = Hemp; Mordanting method: PreM = Pre-mordanting. SM = Simultaneous mordanting. PostM = Post mordanting). *Note*: abcd: Different superscripts at the same row indicate significant differences (*P* < 0.05). If there is the same letter mark, the difference is not significant. If there is a different letter mark, the difference is significant. Values showed the mean of triplicate experiments.

Our results indicated that biomordants could compete with metallic mordants and even exceed them to improve dyeing the fabrics used in this study. We compared color strength and effectiveness of biomardants while dyeing with madder and recycled madder in three mordanting methods and plotted in Fig. 2.

With biomordants in the same mordanting methods, it provided a higher K/S value of recycled madder than madder for dyed cotton and hemp fabrics. For instance, under the methods of simultaneous and pre-mordanting, it showed significantly higher K/S value of dyed cotton by recycled madder with gum rosin than madder as well as with *C. speciosa* in simultaneous and post-mordanting methods (*P* < 0.05). Similarly, simultaneous and pre-mordanting methods presented a better K/S value of madder recycled than madder dyed hemp fabrics with gum rosin (*P* < 0.05). A similar result was obtained out with *C. speciosa* in simultaneous and post-mordanting methods (*P* < 0.05). Generally, it dramatically improves in color strength by recycled madder dyed fabrics with biomordants.

### Colorfastness properties

#### The colorfastness of dyed fabrics with madder

The colorfastness of two fabrics dyed with madder was shown in Table 5. The colorfastness of dyed samples with mordants was higher than without mordant (Indigenous madder dyeing), except that the colorfastness to perspiration and washing were slightly lower. The colorfastness to rubbing in dyed samples with biomordants was 4–5 and 5, and the staining to washing scored 4, 4–5, and 5. The colorfastness of dyed samples to perspiration was generally increased with biomordants 4, 4–5, and 5.

**Table 5:** Colorfastness of the madder dyed two fabrics.

Mordants	Mordanting methods	Washing fastness				Rubbing fastness				Perspiration fastness							
										Acidic				Alkaline			
		CC		CS		Dry		Wet		CC		CS		CC		CS	
		C	H	C	H	C	H	C	H	C	H	C	H	C	H	C	H
Without mordant	- (ID)	1	1	5	4–5	5	3–4	4–5	3–4	3	3–4	4	3–4	3–4	3	4	4
With Gum rosin	Pre-mord	1–2	1–2	4	4–5	5	5	5	4–5	3	3	4	4–5	3–4	2–3	4	4–5
	Simult. mord	2–3	2	4	5	5	5	5	4–5	3	3	4	4–5	3	3	4–5	5
	Post-mord	3	2–3	4	5	5	5	5	5	4	3	4–5	4–5	3–4	3–4	4–5	4–5
With *C. speciosa*	Pre-mord	2	2–3	4	4–5	5	5	5	4–5	2–3	3	4–5	4–5	2–3	4	4–5	4
	Simult. mord	1–2	2	4	5	5	5	4–5	4–5	3	3	4–5	4–5	2–3	2	4	4–5
	Post-mord	2–3	1–2	4	4–5	5	5	5	5	3–4	3	4–5	5	3	2–3	4	4
With Stannous chloride	Pre-mord	1–2	1–2	4	4–5	5	5	5	4–5	4–5	3–4	4–5	4–5	2–3	2–3	4	4
	Simult. mord	1	1–2	4	5	4–5	4–5	5	4–5	2	2–3	3–4	4–5	4	2–3	3–4	4
	Post-mord. (ID)	1–2	1–2	4–5	4–5	4–5	5	5	4–5	3–4	3–4	4–5	4–5	3	3–4	4	4–5
With Alum	Pre-mord	1–2	1–2	4	5	5	5	5	4–5	3	2–3	4	4–5	3	3	4–5	4–5
	Simult. mord	1–2	1–2	4	4–5	5	5	4–5	4–5	4	2–3	4–5	4–5	3	4	4	4–5
	Post-mord. (ID)	2	2	4	5	5	5	5	4–5	4–5	4	4–5	4–5	3–4	4	4	4

CC, Color change; CS, Color staining; C, Cotton; H, Hemp; ID, Indigenous madder dyeing; Pre-mord, Pre-mordanting; Simult. mord., Simultaneous mordanting; Post-mord, Post mordanting.

The results showed that the color change fastness under washing was comparatively higher with mordant than without mordant. In madder dyed cotton with biomordants, color change fastness to washing was better (score 3 in post-mordanting using gum rosin) compared to results without mordants (score 1). Furthermore, it was observed that perspiration fastness was improved to 5 with *C. speciosa* for dyed hemp in the post-mordanting method.

#### The colorfastness of dyed fabrics with recycled madder

The colorfastness of two fabrics with recycled madder-dyed is shown in Table 6. The results indicated that the colorfastness of recycled madder-dyed samples with mordants was strengthened, except for the color change. The colorfastness of recycled madder dyed samples with biomordants to rubbing was 4–5 and 5. Similarly, the color staining in washing and perspiration showed values 4, 4–5, and 5 with biomordant.

**Table 6:** Colorfastness of the recycled madder dyed two fabrics.

Mordants	Mordanting methods	Washing fastness				Rubbing fastness				Perspiration fastness							
										Acidic				Alkaline			
		CC		CS		Dry		Wet		CC		CS		CC		CS	
		C	H	C	H	C	H	C	H	C	H	C	H	C	H	C	H
Without mordant	- (ID)	1–2	1–2	4	4–5	4–5	4–5	4	3–4	3	3–4	4–5	4	3	4	4–5	4–5
With Gum rosin	Pre-mord	1–2	2	4	4–5	5	5	5	4–5	2–3	3	4–5	5	2–3	3	4–5	4
	Simult. mord	2–3	2–3	4–5	4–5	5	4–5	5	5	3	4	4–5	4–5	3	3	4–5	4–5
	Post-mord	3	2–3	4	5	5	5	5	5	3	4	4–5	4–5	4	3–4	4–5	4–5
With *C. speciosa*	Pre-mord	1–2	2–3	4	4–5	5	5	5	5	2–3	3	4–5	4–5	3	4	4	5
	Simult. mord	2	1–2	4	4–5	5	5	4–5	5	2	1–2	4–5	4–5	2	2	4	4–5
	Post-mord	3	3	4	4–5	5	5	5	5	3	4	4–5	4–5	4	3–4	4–5	4–5
With Stannous chloride	Pre-mord	1–2	1–2	4	4–5	5	5	5	5	4	4	4–5	4–5	3–4	2–3	4	5
	Simult. mord	1–2	1–2	4–5	5	4–5	4–5	4–5	5	3	3	4–5	4–5	3–4	3	4–5	4–5
	Post-mord. (ID)	2–3	3	4	5	5	5	5	5	4	3	4–5	4–5	4	3	4–5	4–5
With Alum	Pre-mord	1–2	1–2	4–5	4–5	5	4–5	4–5	5	4–5	3	5	5	3	3–4	4–5	4–5
	Simult. mord	1–2	1–2	4	5	5	4–5	4–5	4–5	3–4	2	4–5	4–5	3	2–3	4–5	4–5
	Post-mord. (ID)	1–2	2	4	4–5	5	5	4–5	5	4	4	4–5	4–5	4–5	3–4	4–5	4–5

CC, Color change; CS, Color staining; C, Cotton; H, Hemp; ID, Indigenous recycled madder dyeing; Pre-mord, Pre-mordanting; Simult. mord., Simultaneous mordanting; Post-mord, Post mordanting.

The results indicated that the color change in washing was improved in samples with biomordants. The color change of dyed cotton fabrics in washing was better (score 3) with biomordants in the post-mordanting method than without mordant (score 1–2).

The color change of dyed two fabrics in washing was improved and scored 3 with *C. speciosa*, while 2 with alum. The experiment results indicated that biomordants were able to enhance the colorfastness in both fabrics dyed with recycled madder.

Tables 5 and 6 showed that the colorfastness of recycled madder dyed fabrics was equal to or slightly higher than the madder dyed fabrics. For instance, the washing fastness of recycled madder dyed fabrics with *C. speciosa* was higher than madder dyed in post mordanting method. The rubbing fastness of recycled madder dyed fabrics with gum rosin was equal to the madder dyed fabrics in post mordanting method. The perspiration fastness of recycled madder dyed fabrics with alum was better than madder dyed fabrics in the pre-mordanting method.

### Phytochemical analysis of extracts

The chromogenic reaction was related to the phenolic hydroxyl and carbonyl groups forming the conjugate system. Both the aqueous extract and the ethyl alcohol extract from the root of *R. wallichiana* have tested their Borntrager reaction. The results suggested that these two extracts showed similar color changes. The ether layer of both extracts was brown, but when NaOH solution was added, the ether layer turned colorless, and the water layer turned red. Their color change indicates that two extracts of *R. wallichiana* contained hydroxyl anthraquinone components. Anthraquinone compounds, which are responsible for the chromophore, are the colorant components of *R. wallichiana* and it was used to give fabric color of yellow to red^57^. Hence, the dyed fabrics showed red and dark yellow colors.

### FTIR and UV–visible analysis

**Figure 3:**
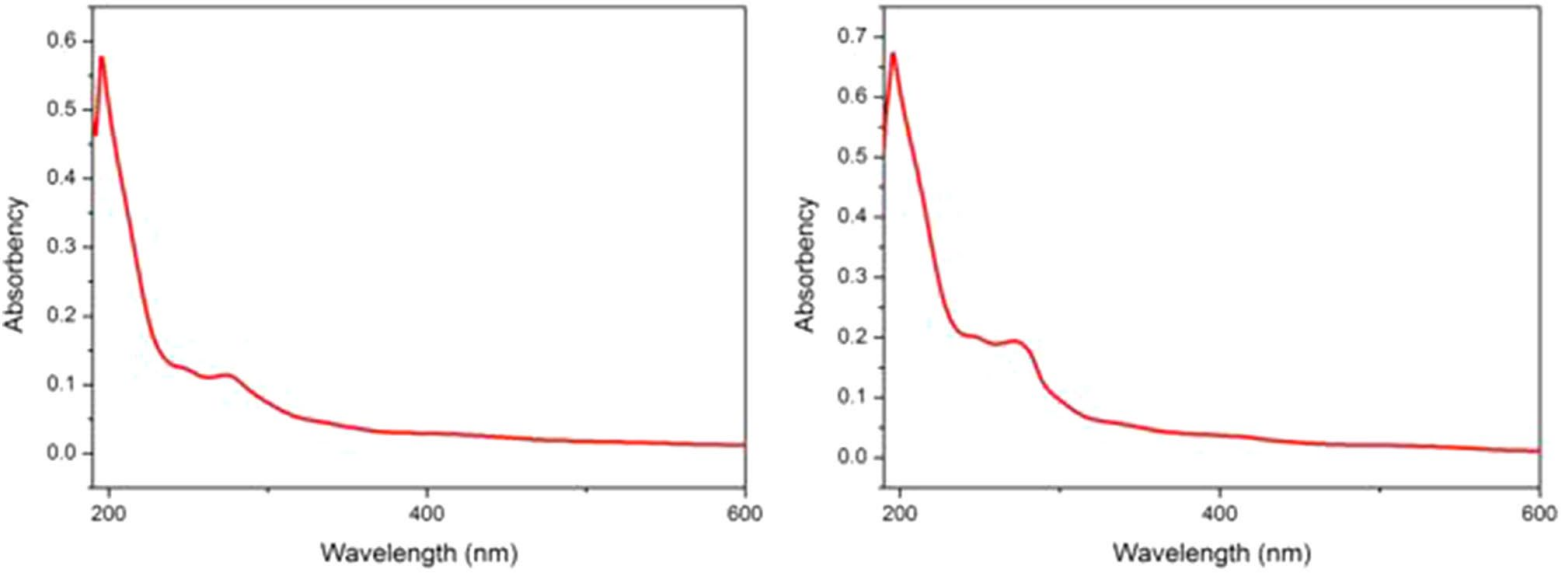
UV–vis spectrum of madder aqueous extraction (left) and madder ethyl alcohol extraction (right).

The UV–visible absorption spectra of madder aqueous extraction and ethyl alcohol extraction were showed in Fig. 3. Both extractions showed similar UV–visible absorption. UV–visible absorption of aqueous extraction showed the peaks (λmax) at 195.50 (0.677), 241.50 (0.204), 259.00 (0.189), and 271.50 (0.195) nm. The absorption of 241.5 nm and 259 nm could be speculated as to the signal of benzene structure. The absorption signal of 271.5 nm was the characteristic signal of quinones. These absorption signals were similar to the UV absorption of anthraquinone components previously reported^58^.

**Figure 4:**
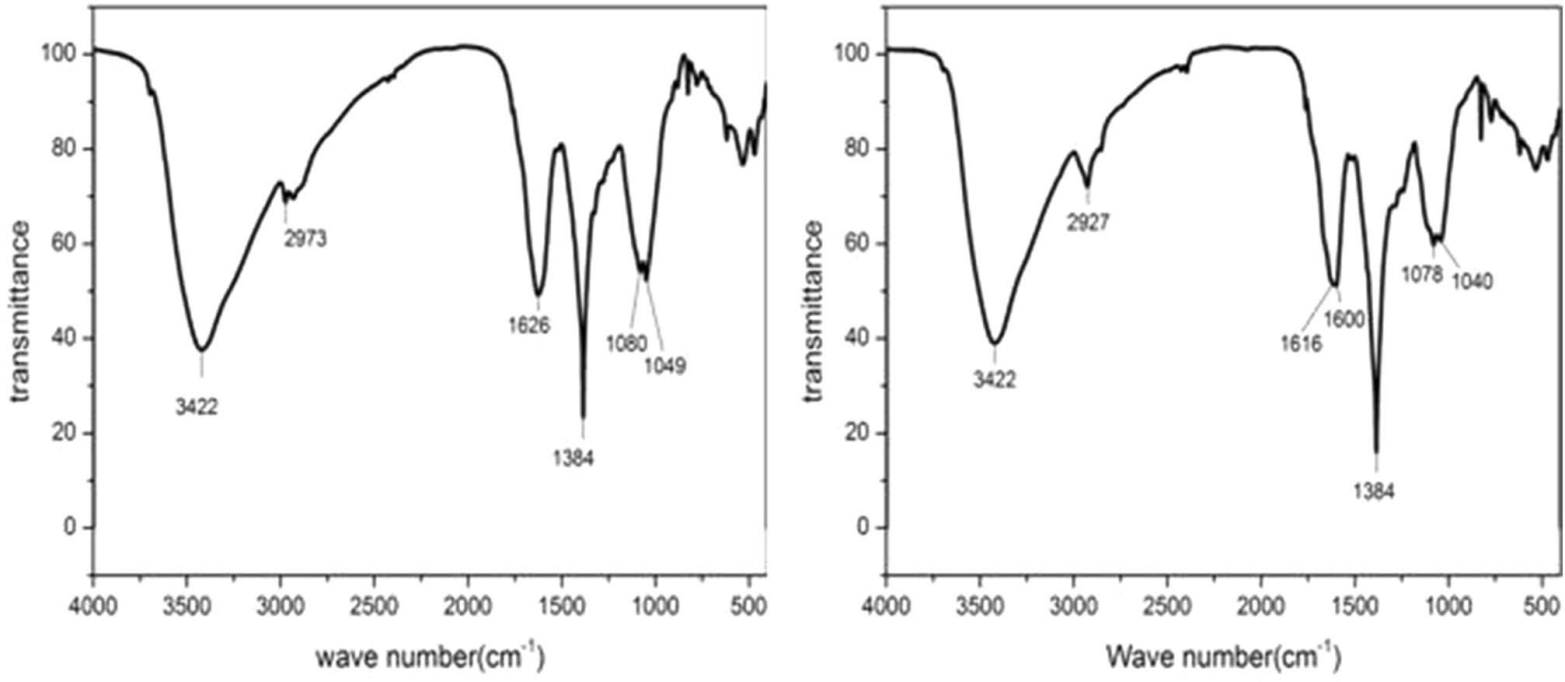
FTIR spectrum of madder aqueous extraction (left) and madder ethyl alcohol extraction (right).

The FTIR analysis results revealed the functional groups in the madder aqueous and ethyl alcohol extract, which were shown in Fig. 4. Two extracts showed similar IR characteristic signals with a broad peak of phenolic -OH group at 3422 cm^−1^. The IR spectra indicated higher absorption in ethyl alcohol extract at wavenumber 3422, 2927, 1616, 1600, 1384, 1078, 1040 cm^−1^. The peak at 1626 cm^−1^ indicated C=O with α-OH, and the peaks at 2973 cm^−1^ and 1384 cm^−1^ indicated C-H stretching in alkanes. The peak at 1600 cm^−1^ corresponds to the C=C stretching of the benzene ring system^59^. The findings of the infrared characteristics suggested that the extract possibly contained anthraquinone components.

## Discussion

### The current status of madder dyeing in Monpa communities

The collection of madder is important in Mêdog County because the Monpa and Tibetan robes are dyed with these. There were 45 key informants in this study, with a majority of women and of the elderly. We found a division of labor regarding dyeing: the collection and extraction of dye plants are done by men, while women do the dyeing, weaving of clothes, and designing patterns. Monpa traditional clothing are mainly worn by older women, especially during festivals. Traditional clothes are not popular among younger people. With economic development, more and more local young people migrate to cities seeking new opportunities. Most of them believe that indigenous madder dyeing will not bring much profit. They do not want to be involved in indigenous madder dyeing due to the arduous and complicated production process. Monpa has no written language. Therefore, traditional knowledge such as madder dyeing is transmitted orally from one generation to another. The following suggestions are made regarding these issues: (1) the existing traditional knowledge about plant dyeing urgently needs to be recorded, collated, and studied urgently by ethnobotanical methods. (2) it is vital to establish a community market for the production, sale of dye plants and related products, stimulating local people to engage in traditional plant dyeing. (3) local governments should provide support for the plant dyeing industry and the research of plant dyeing.

### Evaluation of possible mechanism

**Figure 5:**
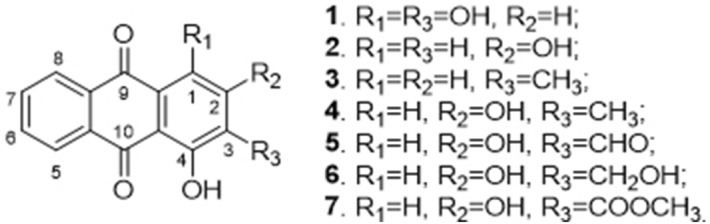
Chemical structures of anthraquinone derivatives found in the roots of *R. wallichiana.*

Since madder (the root of *Rubiaceae*) has little affinity to cellulosics^26^, biomordants could be used as an environmentally enhancer. The main colorants extracted from madder are anthraquinone derivatives (Fig. 5), which are purpurin (**1**), xanthopurpurin (**2**), 1-hydroxy-2-methylanthraquinone (**3**), rubiadin (**4**), nordamnacanthal (**5**), lucidin (**6**), and munjistin methyl ester (**7**)^60–62^. The hydroxy group in the 4-site of anthraquinone derivatives can form a six-membered ring through a hydrogen bond with carbonyl group in the 10-site of the anthraquinone molecule chelated with metal ions^63,64^. However, if R_3_ is a hydroxide radical group, one of the anthraquinone derivatives, the structure of the ortho-dihydroxyl group may enhance the chelation observably^61^. Furthermore, van der Waals forces and intermolecular hydrogen bonds are formed with the hydroxy group in the 4-site and the carbonyl group in the 10-site of anthraquinone derivatives, resulting in the molecule of plant dyes combines with biomordants.

**Figure 6:**
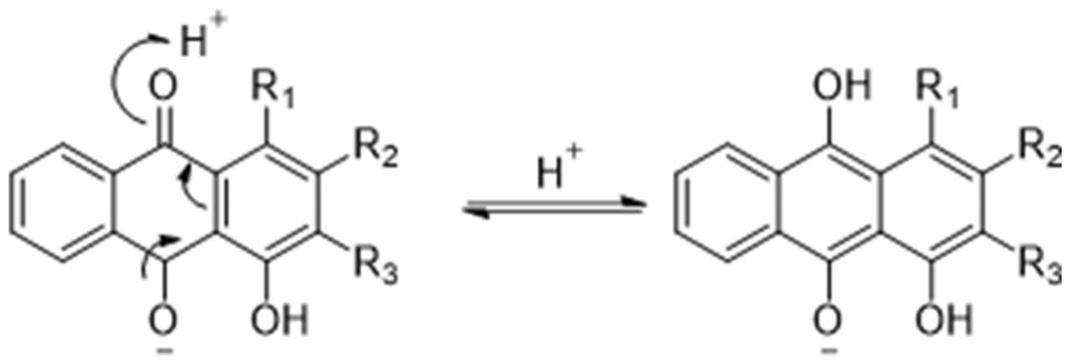
The proposed possible mechanism between *C. speciosa*, cotton/hemp fabrics, and the pigment of *R. wallichiana.*

The possible mechanism between *C. speciosa*, cotton/hemp, and the pigment of *R. wallichiana* is illustrated in Fig. 6. *C. speciose* could help anthraquinone derivatives to form phenol through electron migration because of its acidity. The phenolic form is more likely to react with the cellulose fiber, thus increasing the colorfastness of the dyed cellulose fibers. Also, the fixing mechanism of gum rosin in color properties is attributed mainly to a film's physical properties on fiber surfaces formed by cross-linking. Secondarily to its chemical property of the hydrogen bonding intermolecular forces^42^. Meanwhile, the main component of gum rosin is resin acid (C_19_H_29_COOH)^65^. Its binding sites include carboxyl group and a double bond, forming a chemical bond with anthraquinone derivatives and fibers. As a result, biomordants provide a possible mechanism to apply to the traditional dye standard and improve color properties. This mechanism can also stimulate additional research on the chemical bonds between colored substances and natural fibers. The rational development and application of madder dying and biomordants can lead to innovations in the madder dyed cotton and hemp fabrics industries.

### Cost estimation and prospects

Wastewater from the production of synthetic dyes is harmful to the environment and human health. Hence, it must be treated before discharging into rivers or public water bodies, according to environmental laws in China and elsewhere^20^. This is a significant external cost. On the contrary, sustainable plant dyes can be economically more cost-effective and have a huge potential to be mainstreamed in the textile industry due to saved water treatment costs.

The comparative cost of synthetic and plant dyeing is shown in Table 7. Employing all data, the cost of recycled madder dyeing was lower than madder dyeing by 7.4%. Moreover, the costs of synthetic dyeing were lower than both madder dyeing (34.7%) and recycled madder dyeing (24.8%). Market demand for plant dyes is increasing and becoming more common in production by some high-end textile enterprises^66^.

**Table 7:** Comparative cost of synthetic and plant dyeing for 1 kg fabrics.

Item	Dye type		
	Madder dyeing	Recycled madder dyeing	Synthetic dyeing
Raw materials	29.8 ¥	-	21.3 ¥
Extraction and dyeing process	100.2 ¥	120.0 ¥	68.2 ¥
Additives	5.0 ¥	5.0 ¥	8.5 ¥
Wastewater treatment (m^2^)	–	–	2.2 ¥
Total	135 ¥	125 ¥	100.2 ¥

The estimation of this study showed that 1.2 kg of madder or recycled madder with biomordants were used for 1 kg dyed fabrics through aqueous (indigenous Monpa approaches) or ethyl alcohol (improved by this study) extraction. This improved dye craft could dye more fabrics than indigenous Monpa dye techniques. The use of waste roots could also reduce the cost of materials for madder dyeing. Considering human health and the environment, plant dyeing and recycled plant dyeing are preferable to synthetic dyeing and have significant potential for the textile industry. Although madder and recycled madder dyeing costs are slightly higher than synthetic dyeing, industry production using plant dyes is facing fewer costs of pollution treatment and also can gain market share from green labelling.

It is possible to further reduce the cost of madder and recycled madder dyeing as follows:

1. Improving the dyeing craft can reduce the cost, such as studying the dyeing property of fabric with madder dye concentration;
2. The method of scale cultivation of madder can also be used to save the cost of dye resources to realize the industrialized supply of madder;
3. The residue madder of water extraction could be purchased in some chinese medical factories at a low price. Madder is a valuable medicinal plant in China^67^.

## Conclusion

This study provides valuable information on the possible contribution of Himalayan Monpa traditional knowledge to the textile industry through local plant dye use. The findings bridge a link between traditional knowledge and textile industry development that could lead to an industry production using eco-friendly natural anthraquinone containing dye and biomordants.

Based on the ethnobotanical study of the dye plants of the Monpa community and the evaluation of each dye plant through the CI value, the results show that the CI value of madder is the most significant. We propose utilizing dye residue that is generally abandoned in the indigenous madder dyeing process and residue transformation into a secondary colorant for the textile industry. The innovative dyeing craft with biomordants for cotton and hemp fabrics with madder and recycled madder was established in our research, and the results exhibit good color properties, along with ecological and economic benefits. Dyed fabrics present color variations depending on mordants, mordanting methods, and the types of fabrics.

Our results reveal that the residue from indigenous madder dyeing is a potential source of plant dye for dyeing cotton and hemp fabrics. It can be effectively applied with biomordants for ecological dyeing textiles. An improvement of the traditional dyeing craft could lead to the sustainable use of plant dye and further develop the textile industry in a more sustainable manner.

## Data Availability

All data generated or analyzed during this study are included in this published article and its supplementary information files, which are available from the corresponding author on reasonable request during the current study.
